# Value of CT sinography and analysis of missed diagnosis and misdiagnosis for abdominal wall sinus

**DOI:** 10.1186/s12876-022-02291-0

**Published:** 2022-05-03

**Authors:** Xuechao Du, Yuchang Yan, Pengtao Sun, Shuo Yang, Zhenyu Pan, Sujun Liu, Tao Jiang

**Affiliations:** 1 Department of Radiology, Beijing Chaoyang Hospital, Capital Medical University, 8 Gongren Tiyuchang Nanlu, Chaoyang District, Beijing, 100043 China; 2 Department of Radiology, Beijing Shijitan Hospital, Capital Medical University, Beijing, 100038 China; 3 Department of Hernia and Abdominal Wall Surgery, Beijing Chaoyang Hospital, Capital Medical University, Beijing, 100043 China

**Keywords:** Hernia and abdominal wall surgery, Fistula, Misdiagnosis, Sinography, Computed tomography

## Abstract

**Background:**

The value of CT (computed tomography) sinography in evaluating abdominal wall sinus tracts is currently unclear. The present study aims to investigate the accuracy of CT sinography in diagnosing the extent of abdominal sinus and analyze the reasons for misdiagnosis.

**Materials and methods:**

64 patients with abdominal sinus tract formation (including fistula) undergoing CT sinography in our hospital from January 2018 to November 2020 were retrospectively analyzed. The CT images were blindly and independently re-assessed by two radiologists with 5- and 18-years work experience, respectively. Whether the sinus tract was confined to the abdominal wall or had invaded the abdominal cavity, and whether there was fistula formation were evaluated. The accuracy of CT sinography in diagnosing sinus invasion in the abdominal cavity and fistula formation was calculated. The agreements of CT sinography-surgical results and inter-observer were assessed using weighted-kappa statistics.

**Results:**

The weighted- Kappa of inter-observer agreement (0.825, *P* < 0.001) and CT sinography—surgical results (0.828, *P* < 0.001) were both perfect. The diagnostic accuracy, sensibility, and specificity of sinus tract confined to the abdominal wall were 90.6% (95% CI: 80.7–96.5), 85.7% (95% CI: 67.3–96.0), and 94.4% (95% CI: 81.3–99.3), respectively. The diagnostic accuracy, sensibility, and specificity of fistula formation were 93.8% (95% CI: 84.8–98.3), 89.5% (95% CI: 66.9–98.7), and 95.6% (95% CI: 84.9–99.5), respectively. A total of 4 cases of sinus tract confined to the abdominal wall were misdiagnosed as invading the abdominal cavity, 2 cases of sinus tract invading the abdominal cavity were misdiagnosed as confined to the abdominal wall, 2 cases of enterocutaneous fistula were missed, 1 case of enterocutaneous fistula was misdiagnosed, 1 case of vesico-cutaneous fistula was misdiagnosed, and no cases of vesico-cutaneous fistula were missed.

**Conclusions:**

CT sinography can accurately assess the extent of an abdominal sinus tract and reveal fistula formation, despite some inevitable misdiagnosis and missed diagnosis. Radiologists should find more clues to improve the diagnostic accuracy.

## Introduction

An abdominal wall sinus tract is an infectious fibrous blind channel that leads to the skin, with or without extension into the abdominal cavity. It is a troublesome complication that occurs mainly after hernia and abdominal wall surgery [1]. The occurrence of a sinus tract is related to many factors: surgical factors (i.e., mesh implantation, contaminative incision) [2], postoperative factors (i.e., incision infections, poor drainage) and patient factors (i.e., smoking, obesity, and diabetes) [3, 4]. Without sufficient treatment, the sinus tract can involve the intestine as well as other hollow organs to form a fistula. Its treatment methods vary, depending on the depth of sinus invasion and disease duration. Simple superficial tissue infections can be cured by local drainage or local anesthetic debridement combined with systemic antibacterial drugs; however, if the debridement treatment over-expands, the wound area will be further enlarged and healing will be delayed [5, 6]. For deep sinus tracts, especially when the mesh is infected or the deep abscess does not communicate with the external sinus tract, inadequate conservative treatment may lead to a further deepened sinus tract, increased infection, and even fistula formation. Therefore, it is crucial to accurately assess the depth and extent of sinus invasion and fistula formation before treatment for personalized treatment plans.

CT imaging is a commonly used preoperative examination method [7, 8], which can clearly show the course of the sinus tract and its relationship with surrounding tissues [7], as well as potential causes [9] and important complications. However, CT scans without the use of a contrast agent are of limited value in diagnosing fistula [10, 11], and determine whether a deep abscess is connected to the sinus tract. In recent years, some radiologists injected a contrast agent into the sinus/fistula before CT scans to evaluate anal fistulas [12, 13], and found that CT sinography can reveal all types of anal fistulas and peri-anal fistulas. CT sinography was also used to assess the shape and scope of the sternal sinus, and whether it penetrated the sternum into the mediastinum [14]. However, few studies have been done on the value of CT sinography in assessing abdominal wall sinus tracts. Therefore, the purpose of this study was to explore the accuracy of CT sinography in assessing the extent of sinus invasion, and to analyze the reasons for missed diagnosis and misdiagnosis.

## Materials and methods

### Study population

**Fig. 1:**
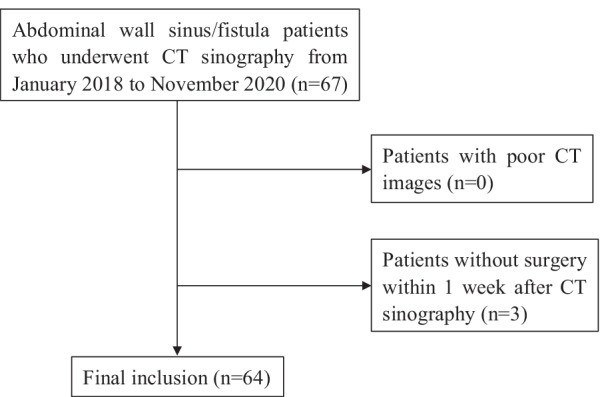
Flowchart of the study population

Figure 1 displays the process of patient selection. Patients with an abdominal wall sinus tract or fistula who underwent CT sinography in our hospital from January 2018 to November 2020 were enrolled in this study. Exclusion criteria were: (a) poor image quality data due to motion artifacts; (b) failure to undergo surgical treatment within 1-week post-CT examination. Ultimately, 64 patients were enrolled (51 males and 13 females) with an average age of 54.9 years (19–84). The study was approved by the Ethics Committee (2020-Scientific Research-462) of our hospital. Informed consent was obtained from all individual participants included in the study.

### CT sinography protocol

An un-enhanced abdominal CT scan was performed on each patient using a 64-section CT scanner (Discovery CT 750 HD, GE Healthcare, Chicago, IL, USA) with the following parameters: kVp, 120; mAs, 80–300; collimation, 128 × 0.6 mm; pitch, 0.6; field of view, 300 mm × 300 mm; slice thickness, 5 mm; slice spacing 5 mm. With the patient in a supine position, the proper amount of a water-soluble non-ionic contrast agent (Iopromide 300, Bayer Schering Pharma AG, Leverkusen, Germany) without dilution was gently injected through a cutaneous opening prior to scanning. Axial and coronal images with a 0.625 mm thickness were reconstructed for further analysis.

### Image analysis

The CT images were blindly and independently re-assessed by two experienced radiologists (XC D and YC Y) with 5- and 18-years work experience, respectively. Whether the sinus tract was confined to the abdominal wall or had invaded the abdominal cavity, and whether there was fistula formation (including enterocutaneous fistula, vesico-cutaneous fistula, etc.) were evaluated.

Diagnostic criteria for sinus tract involvement in the abdominal cavity: the parietal peritoneum adjacent to the sinus tract was discontinuous, and the contrast agent entered the abdominal cavity; or the peritoneal line-like structure was unclear, and the boundary between the intraperitoneal exudation and the sinus was indistinct. The diagnosis criteria for enterocutaneous fistula (ECF): the contrast agent entered the intestine from the sinus tract [15]; or it did not enter the intestine, but air bubbles were detected in the deep infection area of the sinus [16], and the sinus tract had an unclear boundary with the intestine. The diagnostic criteria for vesico-cutaneous fistula: the boundary between the sinus and bladder was indistinct, and density of fluid in the bladder increased due to entry of the contrast agent.

### Statistical analysis

Statistical analysis was performed using SAS version 9.1 software (SAS, Cary, NC, USA) and MedCalc software (MedCalc Software Ltd., Flanders, Belgium). Intraoperative findings (based on methylene blue dye) were used as the gold standard to calculate the accuracy, sensitivity, and specificity of CT sinography in evaluating whether the sinus tract invaded the abdominal cavity (including fistula formation), and whether a fistula occurred. The agreements of CT sinography–surgical results and inter-observer were assessed using weighted-kappa statistics. A Kappa value above 0.81 was considered almost perfect agreement, 0.61–0.80 was considered substantial agreement, 0.41–0.60 was considered moderate agreement, and 0.21–0.40 was considered fair agreement [17]. Statistical significance was defined as *P* < 0.05.

## Results

### Patient characteristics

Of the 64 patients, 43 (67.2%) cases were caused by infection after inguinal hernia repair, 12 (18.8%) cases after ventral hernia (including incision hernia and parastomal hernia) repair, 2 cases after abdominal mass resection, 5 cases after other abdominal cavity surgery, and 2 cases had no clear history of abdominal surgery. According to the intraoperative findings, 28 cases (43.8%) had sinus tract confined to the abdominal wall, 17 cases (26.6%) involved the abdominal cavity without fistula formation, and 19 cases (29.7%) had a fistula. Among the 19 fistula patients, 17 cases had ECF, 1 case had a vesico-cutaneous fistula, and 1 case had combined ECF and vesico-cutaneous fistula formation.

### CT sinography analysis

The weighted- Kappa of inter-observer agreement (0.825, *P* < 0.001) and CT sinography—surgical results (0.828, *P* < 0.001) were both perfect. The diagnostic accuracy of sinus tract invasion of the abdominal cavity was 90.6% (Table 1), and that of fistula formation was 93.8% (Table 2). Diagnostic accuracy was 95.3% for ECF, and 98.4% for vesico-cutaneous fistula.

**Table 1:** Evaluation of CT sinography on the extent of sinus tract invasion

CT	Operation		Acc (95% CI)	Sen (95% CI)	Spe (95% CI)	LR+ (95% CI)	LR− (95% CI)
	Abdominal wall (28)	Abdominal cavity (36)					
Abdominal wall	24	2	90.6% (80.7–96.5)	85.7% (67.3–96.0)	94.4% (81.3–99.3)	15.4 (4.0–59.8)	0.2 (0.1–0.4)
Abdominal cavity	4	34					

*Acc* accuracy, *CI* confidence interval, *Sen* sensitivity, *Spe* specificity, *LR*+ Positive likelihood ratio, *LR*− Negative likelihood ratio

**Table 2:** Evaluation of CT sinography on fistula formation

CT	Operation		Acc (95% CI)	Sen (95% CI)	Spe (95% CI)	LR+ (95% CI)	LR− (95% CI)
	Fistula (19)	No fistula (45)					
Fistula	17	2	93.8% (84.8–98.3)	89.5% (66.9–98.7)	95.6% (84.9–99.5)	20.1(5.2–78.7)	0.1(0.0–0.4)
No fistula	2	43					

*Acc* accuracy, *CI* confidence interval, *Sen* sensitivity, *Spe* specificity, *LR*+ Positive likelihood ratio, *LR*− Negative likelihood ratio

### Missed diagnosis and misdiagnosis cases of CT sinography (Table 3)

**Fig. 2:**
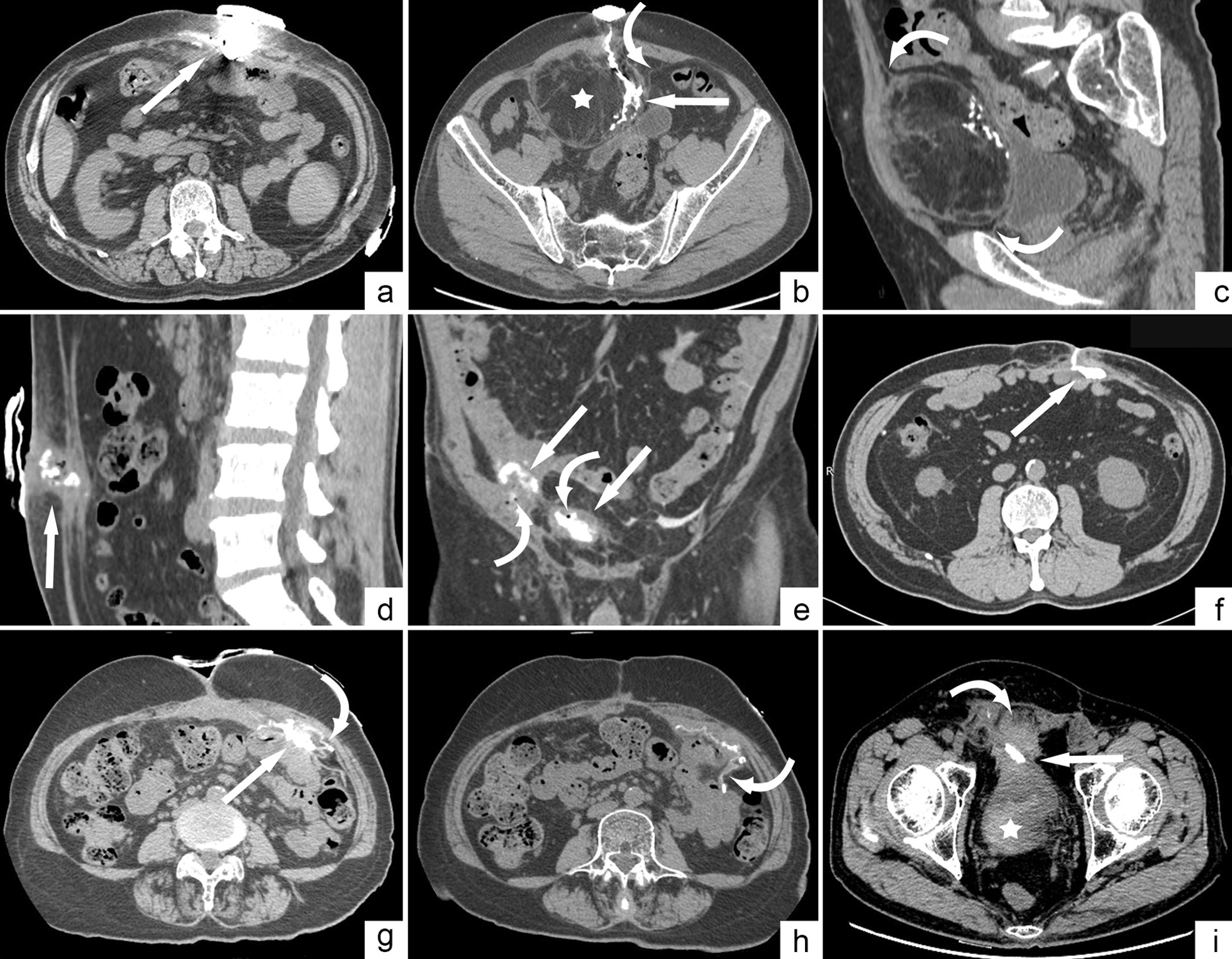
Missed diagnosis and misdiagnosis cases of CT sonography. **a** Case 1 (sinus confined to the abdominal wall), a 56-year-old male, CT shows that the abscess cavity protrudes into the abdominal cavity (arrow). **b**, **c** Case 2 (sinus tract confined to the abdominal wall), a 65-year-old male, the sinus runs in a strip antero-posteriorly (arrow), and a mass formed by fat necrosis (☆) can be seen in the adjacent abdominal wall. The adjacent peritoneum appears intact (curved arrow). **d** Case 5 (sinus involving the abdominal cavity), a 66-year-old man, the sinus is mainly located in the abdominal wall on CT image (arrow). The adjacent peritoneum is thickened, but its integrity could not be judged. **e** Case 6 (sinus involving the abdominal cavity), a 71-year-old man, the sinus involves the abdominal cavity, with deep abscess (arrow) containing many small bubbles (curved arrow), leading to misdiagnosis of ECF. **f** Case 7 (ECF), an 82-year-old female. Most of the lesion is in the abdominal wall (arrow) and closely adhered to the adjacent intestinal wall. There are no signs of ECF. **g**, **h** Case 8 (ECF), a 64-year-old male, the sinus involves the abdominal cavity, and the adjacent mesh is thickened and curled (curved arrow). No signs of ECF are found. **i** Case 4 (sinus confined to the abdominal wall), a 32-year-old female, the abdominal wall around the sinus is obviously thickened (curved arrow), and the border with the anterior bladder wall is indistinct (arrow). The bladder density increases up to 76HU due to entry of the contrast agent (☆), which lead to a misdiagnosis of vesico-cutaneous fistula. *CT* computed tomography, *ECF* enterocutaneous fistula

In four cases (Cases 1–4), the anteroposterior diameter of the lesion was significantly larger than the thickness of the adjacent abdominal wall, and CT sinography diagnosed the sinus tracts as involved in the abdominal cavity; however, surgery confirmed that the parietal peritoneum was intact and the lesions were confined to the abdominal wall (Fig. 2a–c). Two cases were misdiagnosed as confined to the abdominal wall, but surgery confirmed that the lesions penetrated the abdominal cavity: one case (Case 5) involved the greater omentum (Fig. 2d), and the other case (Case 7) adhered to the adjacent bowel, forming an ECF (Fig. 2f).

**Table 3:** Misestimation cases of CT sinus angiography

Cases	Gender	Age	Surgery history	Mesh	Comorbidity	CT evaluation	Intraoperative finding
1	Male	56	Inguinal hernia repair	PP flat mesh	Diabetes	Cavity	Wall
2	Male	65	Inguinal hernia repair	Self-Gripping PP Mesh	Hypertension	Cavity	Wall
3	Male	66	Inguinal hernia repair	PP flat mesh + plug	No	Cavity	Wall
4	Female	32	Inguinal hernia repair	PP flat mesh + plug	No	VCF	Wall
5	Male	40	No relative surgery	No	No	Wall	Cavity
6	Male	71	Inguinal hernia repair	PP flat mesh	No	ECF	Cavity
7	Female	82	Parastomal hernia repair	PP + PLCL composite mesh	Hypertension	Wall	ECT
8	Male	64	Laparotomy for ileus	PTFE	No	Cavity	ECF

*Cavity* abdominal cavity, *Wall* abdominal wall, *ECF* enterocutaneous fistula, *VCF* vesico-cutaneous fistula

Ten cases were diagnosed with ECF based on the appearance of contrast agent in the intestine, and all were confirmed via operation. Seven patients were diagnosed with ECF according to free air bubbles in the deep infection area of the sinus tract, of which 6 cases were confirmed to be ECF during surgery, and 1 case (Case 6) had no internal fistula formation (Fig. 2e).

Two cases (Cases 7 and 8) showed adhesion between the deep part of the sinus tract and intestine, and neither free gas bubbles in the infection foci nor contrast agent in the intestine were detected. Therefore, CT sinography failed to detect ECF formation. However, during operation, 0.5–1.0 cm sized intestinal wall defects were found after careful separation of the adhesions (Fig. 2f–h).

One case (Case 4) showed obvious adhesion between the deep part of the sinus tract and bladder wall via CT sinography. Although no bladder wall defect was clearly seen, density of the fluid in the bladder increased to 76HU due to the presence of the contrast agent, and the diagnosis of vesico-cutaneous fistula was reached. However, since the bladder wall was still intact after separating the adhesion (Fig. 2i), there was no misdiagnosis of vesico-cutaneous fistula. Furthermore, there was one other patient whose bladder, as well as bilateral renal pelvis and ureter, was filled with obvious high attenuation (up to 650HU), indicating that the contrast agent in the bladder was excreted from the kidneys, rather than the fistula. Therefore, this patient was not diagnosed with vesico-cutaneous fistula.

## Discussion

In our investigation, we used CT sinography to evaluate the abdominal wall sinus tract and found that CT sinography had high diagnostic accuracy in evaluating the extent of abdominal wall sinus tracts and fistula presence. Furthermore, we analyzed cases in which CT evaluation results and surgical findings were inconsistent, and summarized possible reasons for the discrepancies.

### Whether or not sinus tracts invade the abdominal cavity

The criterion for diagnosing lesion invasion into the abdominal cavity is whether the parietal peritoneum, which separate the abdominal wall and abdominal cavity, is intact. Since the parietal peritoneum is very thin, it is hardly visualized on CT images, or only appears as a fine, thin line-like structure [18]. The sinus tract can cause peritonitis, which is characterized by a thickened peritoneum [18]. When the sinus tract breaks through the peritoneum but only involves a small amount of the omentum or mesenteric, it is difficult to diagnose abdominal cavity involvement by CT. When judging lesion involvement in the abdominal cavity, it is important to carefully observe whether the adjacent peritoneum is complete and continuous. Although the majority cannot be distinguished, one case in this study could have avoided misdiagnosis had careful observation taken place. In this case (Fig. 2b, c), due to the formation of a large mass caused by fat liquefaction and necrosis in the abdominal wall, the anteroposterior diameter of the sinus tract was significantly larger than the thickness of the adjacent abdominal wall. As a result, abdominal cavity involvement was initially diagnosed via CT. However, after retrospectively observing the CT images and discovering the line-like peritoneum around the sinus tract (Fig. 2b, c curved arrow), we found that the abdominal cavity was not involved.

### Missed diagnosis and misdiagnosis analysis of ECF

An ECF is defined as an abnormal communication between the gastrointestinal tract and skin. Management of ECF is a complex problem [19]. Therefore, it is essential to determine whether the sinus tract involves in the intestine. Unlike an intestinal fistula caused by acute gastrointestinal perforation, in which free intra-peritoneal air can be detected, an ECF caused by an abdominal wall sinus tract is difficult to diagnose by a conventional CT scan, and sometimes it is not easy to distinguish from the fistulas which drain intra-abdominal collection [15]. In this study, when using CT sinography to diagnose ECF, the diagnostic accuracy was 95.3%, with two missed cases and one misdiagnosed case.

The direct diagnostic sign of ECF from CT sinography is entry of the injected contrast medium through the sinus into the intestine. In our study, the false-positive rate of this diagnostic standard was zero, but the false-negative rate was rather high. Reasons for the missed diagnosis of ECF include: (1) the non-absorbable mesh implanted during hernia repair erodes in the intestine [5] and adheres tightly; (2) The intestinal wall defect area is surrounded by inflammatory tissue and does not communicate with the sinus tract; (3) The origin of the fistula may be edematous or clogged with necrosis, thus preventing the contrast agent from entering the intestine [20, 21]; (4) The injection volume is insufficient due to the patient's pain and other discomfort; (5) The injection pressure is insufficient, in order to avoid unnecessary damage (such as causing false passages) [22]. To make up for missed diagnoses due to the latter three reasons, we also used an important indirect sign when diagnosing ECF, that is, the presence of air bubbles in the deep area of the sinus tract, which was caused by gas leakage in the defect area of the intestinal wall [16]. This indirect sign effectively improved the accuracy of ECF diagnosis, but it also led to a false-positive case. We speculated that, the gas may have been formed by gas-forming bacteria, or entered through the opening of the sinus tract. For cases where the bowel defect is surrounded by inflammatory tissue, hindering the communication between the damaged bowel and the sinus tract, CT enterography may improve the diagnostic accuracy. It may feature as the diffusion of high attenuation contrast agent out of the intestine lumen.

### Misdiagnosis analysis of vesico-cutaneous Fistula

In this study, one patient was misdiagnosed with a vesico-cutaneous fistula due to increased fluid attenuation in the bladder, manifesting the entry of the contrast agent. The presence of contrast agent in the bladder was possibly formed by the concentration of blood containing a small amount of contrast agent through the kidneys. And the agent may enter the blood through surrounding micro-vessel and peritoneum (which has strong absorption capacity [23]). Inflammatory cells in the sinus tract wall can produce vascular endothelial growth factor, which leads to an increase in tiny blood vessels and increased permeability of the blood vessel wall. Moreover, micro-vessel around the sinus tract may be damaged by inflammation or iatrogenic procedures, making it easier for the contrast agent to enter the blood. Thus, to avoid the misdiagnosis of vesico-cutaneous fistula, we recommend shortening the time interval between CT imaging and contrast agent injection. Furthermore, in patients with ambiguous diagnosis, CT urography may be helpful in ruling out the fistula.

## Conclusion

In summary, CT sinography can accurately assess the extent of abdominal wall sinus tract invasion and detect serious complications such as fistula formation, despite some inevitable misdiagnosis and missed diagnosis. Radiologists should find more clues to improve the diagnostic accuracy.

## Data Availability

The datasets generated and/or analyzed during the current study are not publicly available due our hospital’s restrictions but are available from the corresponding author on reasonable request.

## Abbreviations

CT: Computed tomography
ECF: Enterocutaneous fistula
CI: Confidence interval
